# Echinacoside exhibits antidepressant-like effects through AMPAR–Akt/ERK–mTOR pathway stimulation and BDNF expression in mice

**DOI:** 10.1186/s13020-021-00549-5

**Published:** 2022-01-05

**Authors:** Han-Wen Chuang, Tse-Yen Wang, Chih-Chia Huang, I-Hua Wei

**Affiliations:** 1 Graduate Institute of Biomedical Sciences, China Medical University, Taichung, Taiwan; 2 Department of Post-baccalaureate Chinese Medicine, China Medical University, Taichung, Taiwan; 3 Tsaotun Psychiatric Center, Ministry of Health and Welfare, Nantou, Taiwan; 4 Department of Psychiatry, China Medical University, Taichung, Taiwan; 5 Department of Psychiatry, China Medical University Hospital, Taichung, Taiwan; 6 Program in Translational Medicine, National Chung Hsing University, Taichung, Taiwan; 7 Department of Anatomy, China Medical University, Taichung, Taiwan

**Keywords:** AMPAR, Akt, ERK, mTOR, Echinacoside, Antidepressant

## Abstract

**Background:**

Several natural products have been demonstrated to be effective in the treatment of depressive disorders. Echinacoside, a naturally occurring phenol extracted from *Cistanche tubulosa*, *Echinacea angustifolia*, and *Cistanche* spp, has a wide range of physiological effects, such as antioxidation, neuroprotection, anti-inflammatory, and immunoregulation, which are closely related to depression. In addition, echinacoside can activate protein kinase B (Akt), extracellular signal–regulated kinase (ERK), and brain-derived neurotrophic factor (BDNF) in the brain. A key downstream event of the Akt, ERK, and BDNF signaling pathways, namely mechanistic target of rapamycin (mTOR) signaling, plays a crucial role in generating an rapid antidepressant effect. Thus, echinacoside is a promising therapeutic agent for depression. However, research regarding the role of echinacoside in antidepressant effect and brain mTOR activation remains lacking.

**Materials and methods:**

The forced swimming test and Western blot analysis in C57BL/6 mice was used to investigate the antidepressant-like activities of echinacoside and the underlying mechanism involved inα-amino3-hydroxy-5-methyl-4-isoxazolepropionic acid receptor (AMPAR)–Akt/ERK–mTOR pathway.

**Results:**

We confirmed the suggestions by previous reports that echinacoside activates Akt/ERK signaling and further demonstrated that echinacoside could provide antidepressant-like effects in mice via the activation of AMPAR–Akt/ERK–mTOR pathway in the hippocampus.

**Conclusions:**

To the best of our knowledge, our study is the first to reveal that echinacoside is a potential treatment for depressive disorders. Moreover, the present study suggests a mechanism for the neuroprotective effect of echinacoside.

**Supplementary Information:**

The online version contains supplementary material available at 10.1186/s13020-021-00549-5.

## Introduction

Major depressive disorder is a common and serious psychiatric disorder which exacts significant personal, family, and social burden and becomes the leading cause of disability worldwide by 2020 [1] . The actions of current treatments for depression depend on increases of monoamine neurotransmitters (namely dopamine, norepinephrine, and serotonin) via block one or more of the monoamine reuptake. But, only 50% of patients who receive current treatments recover within the first 6 months, and their recovery rate declines sharply over time and many patients continue to have depressive episodes even with regular treatment [2]. Meanwhile, current monoamine-based antidepressants require several weeks to provide therapeutic effects [2]. The time lag and lack of efficacies of current antidepressants are serious problems, particularly for those depressed patients with suicide risk. And, it is an urgent matter to develop a new-generation antidepressant with fast and effective properties. Recently, ketamine, an *N*-methyl-d-aspartate receptor (NMDAR) antagonist, was unexpectedly found to have a rapid and long-acting antidepressant effect following a single subanesthetic dose [3, 4]. Subsequent preclinical studies extensively investigate the rapid antidepressant mechanism of ketamine. These studies indicated that when the glutamate NMDAR is blocked by ketamine, glutamate stimulates another receptor—α-amino3-hydroxy-5-methyl-4-isoxazolepropionic acid receptor (AMPAR) more; then activates downstream protein kinase B (Akt)/extracellular signal–regulated kinase (ERK) signal transduction cascades followed by the mechanistic target of rapamycin (mTOR) activation [5, 6]. Later, several studies suggested the rapidly activated AMPAR–Akt/ERK–mTOR pathway may be a convergent mechanism of antidepressants with fasting-acting properties [7]. Thus, other agents that can trigger the biochemical cascades responsible for the fasting-acting antidepressant effect of ketamine, namely AMPAR–Akt/ERK–mTOR pathway, may also have rapidly antidepressant potential.

Echinacoside, a naturally occurring phenol isolated from *Cistanche tubulosa*, *Echinacea angustifolia*, and *Cistanche* spp, has been used as a traditional herbal medicine in Chinese and Western countries for its antioxidation, neuroprotection, anti-inflammatory, antitumor, antiaging, hepatoprotection, immunoregulation, and learning memory improvement effects [8]. The activation of the hippocampus phosphoinositide 3-kinase (PI3K)–Akt pathway has been found to be a key mechanism underlying the memory enhancement effect in rats with Alzheimer-like disease [9]. In addition, the Trk–ERK pathway activation and BDNF accumulation induced by echinacoside prevents injuries caused by rotenone [10].

Based on the prediction that the activations of Akt and ERK induced by echinacoside will activate their downstream target mTOR signaling [6], echinacoside may exhibit rapid antidepressant effects. However, the antidepressant effects of echinacoside remain unexplored and the effects of echinacoside on brain Akt/ERK downstream mTOR signaling are also unclear. Forced swimming test (FST) is the most used animal test for predicting antidepressant effects [11, 12]. Li et al. found a single dose of ketamine provided acute antidepressant-like effect in FST accompanied with increased activated mTOR signal [6]. And, the antidepressant-like actions observed in FST were blocked by AMPAR inhibitor NBQX and mTOR inhibitor rapamycin [6, 13]. The same effects are also noted in several preclinical studies [7]. Therefore, we tested our hypothesis by investigating the acute antidepressant-like effects of echinacoside in the FST and related changes of Akt, ERK, mTOR activations, and BDNF expression following echinacoside treatment. In addition, using pharmacological inhibitors, we also examine whether the antidepressant-like effects of echinacoside are depended on the AMPAR, Akt, ERK, and mTOR activations. The effects of echinacoside on the phosphorylated Akt, ERK, mTOR, and BDNF expression were further examined with or without inhibitors.

## Materials and methods

### Animals

Male C57BL/6 mice aged 6–8 weeks and weighing 23–25 g were adapted to our laboratory animal center for at least 7 days before the experiment. The mice were maintained in a controlled environment with 23 ℃ ± 1 ℃ temperature, 55% ± 5% humidity, 12-h light/dark cycle, and ad libitum food and water. The mice were transferred to the experimental room for subsequent studies. All experiments were approved by the Institutional Animal Care and Use Committee of China Medical University, Taiwan (permit No. CMUIACUC-2016-072-1).

### Drug administration

Mice were treated with echinacoside (Sigma Aldrich), desipramine (Sigma Aldrich), MK2206 (MedChemExpress), NBQX (Sigma Aldrich) dissolved in 0.9% saline, SL327 (Sigma Aldrich), and rapamycin (Toku-E) dissolved in 0.5% ethanol through an intraperitoneal injection of 0.01 mL/g body weight. The mice in the dose-dependent group were given normal saline, desipramine (20 mg/kg, a tricyclic antidepressant as a positive control) [14], and echinacoside (20, 30, or 40 mg/kg) 30 min before the FST. The other group was treated with the mTOR inhibitor rapamycin (20 mg/kg) [15] or AMPAR inhibitor NBQX (30 mg/kg) [16] 30 min before echinacoside (30 mg/kg, ideal dosage derived from the dose-dependent group) or saline and the Akt inhibitor MK2206 (60 mg/kg) [17] or ERK inhibitor SL327 (40 mg/kg) [18] 1 h before echinacoside or saline administration.

### Forced swim test

The FST is a rodent behavioral test adopted to evaluate the efficacy of antidepressant drugs, and the index is immobility time [11, 12]. First, we conducted a 15-min swim procedure 24 h before the 5-min formal FST in a transparent cylindrical tank with water, in accordance with previous reports. The formal FST was executed 30 min after the injections. We deposited the experimental mice in a cylinder (height: 40 cm; diameter: 20 cm) filled with water (height: 10 cm) at 25 °C for 5 min and recorded their behavioral performance by using a digital camera. The immobility periods were quantified automatically by using EthoVision.

### Open field test

We used an open field test (OFT) to analyze the locomotor ability of mice for excluding the possibilities of false-positive results in the FST [12]. The mice were placed into a plastic cage with a 60 cm × 60 cm board and surrounded by 50-cm-high walls; subsequently, the mice were allowed to explore the cage for 5 min after drug administration. Mice movements were recorded using a digital camera, and the total movement in the 5-min period was computed using EthoVision.

### Animal tissue preparation for Western blot analysis

After the OFT was conducted, four mice were sacrificed and their hippocampus removed and dried with nitrogen for further Western blot analysis. The collected tissues were stored at − 80 °C until use. Subsequently, 250 µL of lysis buffer was added per tube for the grinding of the collected tissues. The mixture was centrifuged at 15,000 rpm for 15 min at 4 °C after grinding. The supernatant was transferred to new centrifugal tubes and heated for 7 min with sample buffer. After cooling down the mixture by using a cooling machine set at 4 °C, we preserved all samples at − 80 °C. Subsequently, we conducted Western blot analysis to measure the immunoreactions of proteins in the hippocampus. Previously described procedures for Western blotting were followed [15]. The details are provided in Additional file 1.

### Data analysis

SPSS 12.0 was used for statistical analysis. The data of behavioral experiments were measured using one-way analysis of variance followed by Tukey post hoc tests. Variations in Western blot analysis were analyzed using the nonparametric Mann–Whitney test to compare the two groups. All results were considered significant at *P* < 0.05, and the tests were two-tailed.

## Results

### Effects of echinacoside on immobility during the FST and on phosphorylations of mTOR, Akt, and ERK levels

**Fig. 1:**
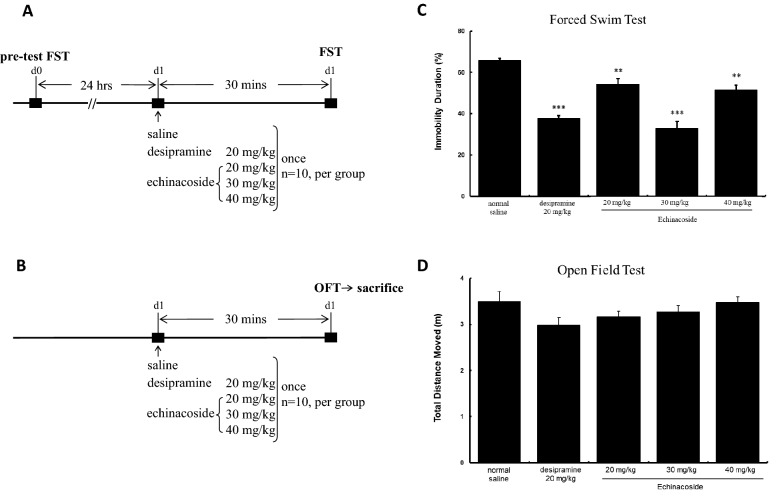
Experimental mice treated with saline, desipramine (20 mg/kg, a tricyclic antidepressant as a positive control) and three different doses of echinacoside (20, 30 or 40 mg/kg) by intraperitoneally injection then detected behavior by forced swimming test (FST) (**A**) or open field test (OFT) (**B**). Mice were respectively administrated by normal saline, desipramine (20 mg/kg) and echinacoside (20, 30 or 40 mg/kg) 30 min before FST and 15-min pre-swimming were conducted at 24 h before the experiment (**A**). The percentage of immobility time significantly reduced in the groups of three different doses of echinacoside and desipramine-treated group (**C**) (ANOVA, F(4,45) = 32.454, *p *< 0.001, n = 10 per group). Mice were injected by normal saline, desipramine (20 mg/kg) and echinacoside (20, 30 or 40 mg/kg) 30 min before OFT (**B**). The total distance moved linked to the animals’ locomotor activity ability showed no difference between saline and all treating groups (**D**) (ANOVA, F(4,45) = 1.859, *p*> 0.05, n = 10 per group). (**p < 0.01, ***p < 0.001 compared with saline-treated group with Tukey *post hoc* analysis); values shown are mean ± SEM

FST, the most used animal test for predicting antidepressant effects [11, 12], was applied to measure the antidepressant-like effects of echinacoside in mice (Fig. 1A). Furthermore, the OFT was used to evaluate the possibilities of false-positive results in the FST [12] (Fig. 1B). Echinacoside at the doses of 20, 30, and 40 mg/kg significantly reduced the immobility of mice by 17.5%, 50.0%, and 21.5% compared with control mice (Fig. 1C). And, a single injection of the positive-control drug, desipramine (20 mg/kg), significantly reduced immobility. Echinacoside at any of the tested doses and desipramine did not influence the locomotor activity of mice (Fig. 1D). The results indicate echinacoside generates antidepressant-like effects. In addition, an inverted U-shaped dose–response relationship was observed in the decreased immobility in the FST following echinacoside treatment.

**Fig. 2:**
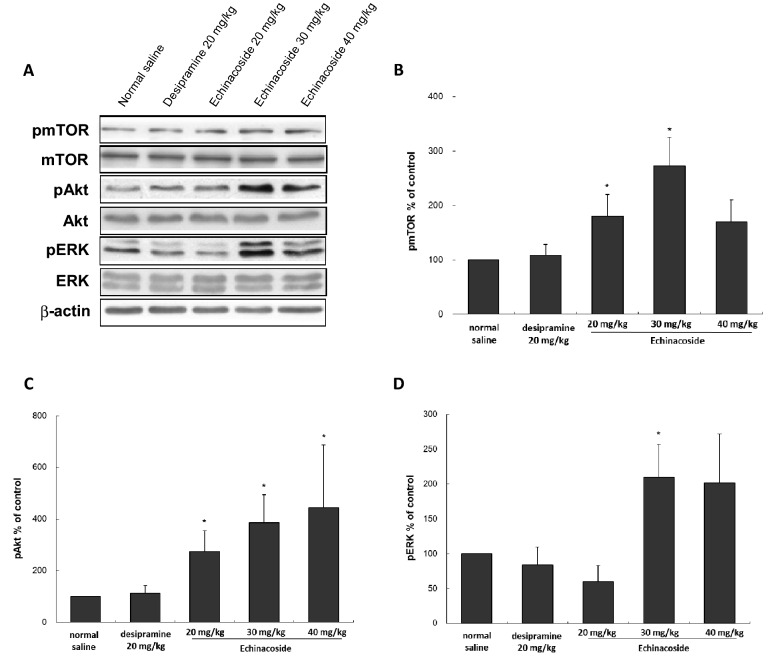
Effects of different echinacoside doses (20 mg/kg, 30 mg/kg or 40 mg/kg) and desipramine (20 mg/kg) intraperitoneally injected on the phosphorylation of mTOR, Akt, and ERK in the hippocampus of mice. Western blot analysis of phosphorylation of mTOR, Akt and ERK were conducted (**A**). The densitometry analyses of the blot (normalized to β-actin) verify the enhanced activity of pmTOR in group treated with echinacoside at 20 mg/kg and 30 mg/kg, not in groups treated with echinacoside at 40 mg/kg and desipramine (**B**). The increased pAkt in the mice hippocampus following treatment with echinacoside were showed in a dose-dependent manner (**C**). Only echinacoside at 30 mg/kg significantly increased pERK (**D**). Total levels of mTOR, Akt, and ERK were not different among the groups. (*p < 0.05 compared with saline-treated group by Mann-Whitney *U* test); n = 4 per group, values shown are mean ± SEM

Based on the previous studies demonstrating that echinacoside can increase Akt [9] and ERK [10] signaling and the fast-acting antidepressant effects of ketamine depend on Akt/ERK downstream mTOR activation [6], we study whether echinacoside would also increase activated Akt, ERK, and related downstream signaling protein mTOR. Using Western blot analysis, the intensities of the activated Akt, ERK, and mTOR were evaluated following echinacoside treatment (Fig. 2 A). As showed in Fig. 2B, the increased mTOR activation was also U-shaped dose-dependent, as the post hoc test revealed that echinacoside at relatively low doses (20 and 30 mg/kg), but not at a higher dose (40 mg/kg; Fig. 2B) increased activated mTOR. The trend was consistent with the decreased immobility in the FST. However, the U-shaped dose–dependent relationship was not observed in Akt and ERK activations, we found that all doses (20, 30, and 40 mg/kg) of echinacoside significantly increased pAkt level in mice (Fig. 2C). In addition, echinacoside at 30 mg/kg resulted in significant increases in pERK levels, but the lower (20 mg/kg) and higher doses (40 mg/kg) did not have a significant influence (Fig. 2D). Traditional antidepressant desipramine did not significantly influence pmTOR, pAkt, and pERK levels.

We found that echinacoside at 30 mg/kg could provide antidepressant effects with significant increases in the intensities of pmTOR, pAkt, and pERK. Therefore, we used echinacoside at 30 mg/kg for all further experiments.

### Effects of AMPAR inhibitor NBQX and mTOR inhibitor rapamycin on the decreased immobility caused by echinacoside in the mice FST

**Fig. 3:**
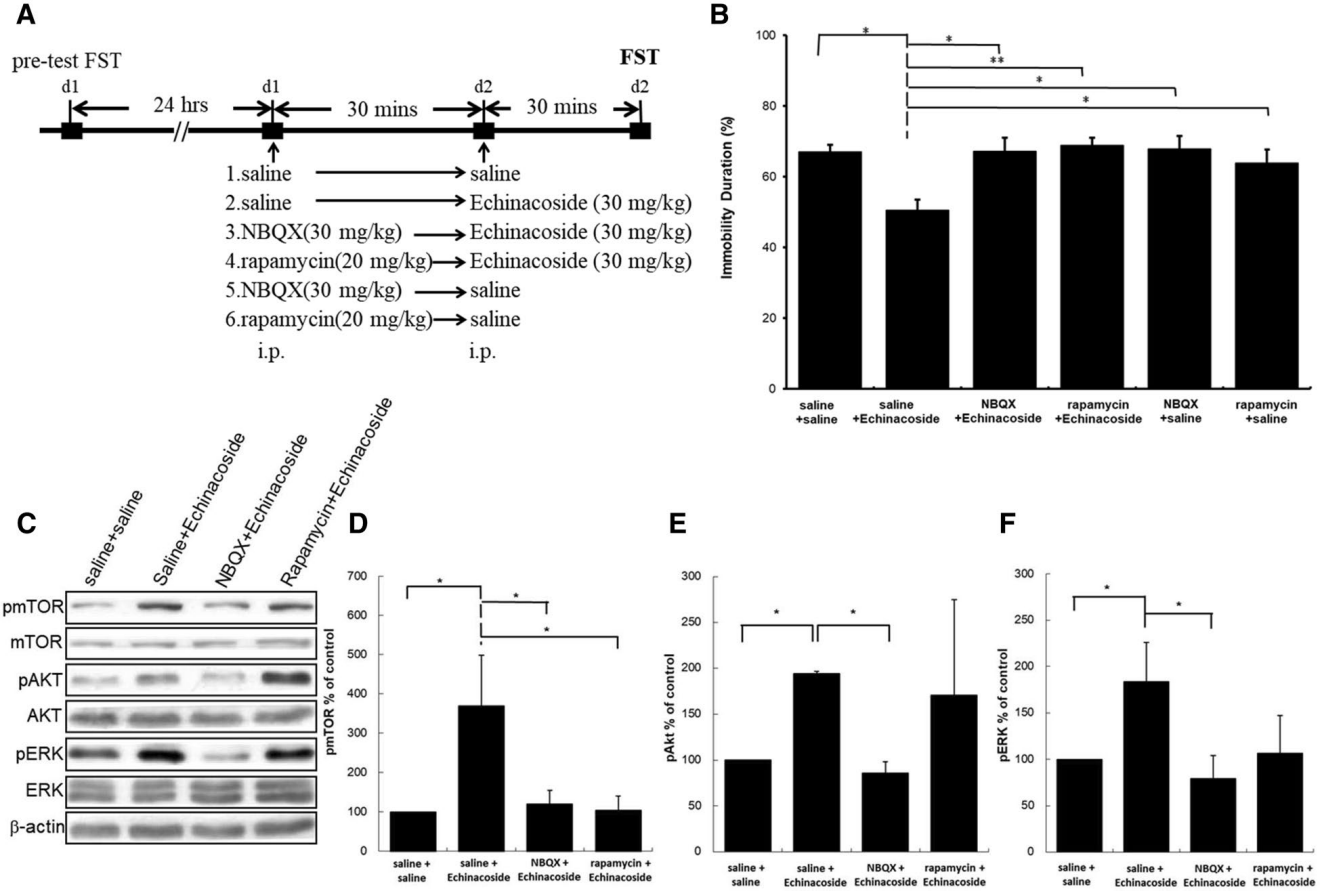
Effects of NBQX (30 mg/kg) or rapamycin (20 mg/kg) on the immobility duration in forced swim test (FST) and Western blotting of pmTOR, pAkt, and pERK from hippocampus of echinacoside (30 mg/kg)-treated mice. The timeline exhibits the experimental procedure under administration of drugs (**A**). Statistically analysis showed that the effect of reduction of immobility duration in FST resulted from echinacoside treatment is inhibited when the mice were pretreated with NBQX and rapamycin (**B**) (ANOVA, F(5.54) = 4.501, p < 0.01; n = 10 per group; *p < 0.05, **p < 0.01 compared with saline-treated group with Tukey *post hoc* analysis). Western blot analysis of phosphorylation of mTOR, Akt, and ERK was performed (**C**). The densitometry analyses of the blot (normalized to β-actin) confirmed the increased activity of pmTOR (**D**), pAkt (**E**), and pERK (**E**) in the echinacoside administrated group. The increased expression of pmTOR (**D**), pAkt (**E**), and pERK (**F**) resulted from echinacoside treatment being blocked when mice were pretreated with NBQX. The increased expression of pmTOR (**D**) resulted from echinacoside treatment is blocked when mice were pretreated with rapamycin and the increased expression of pAkt (**E**) and pERK (**F**) resulted from echinacoside treatment is not blocked; n = 4 each group; * *p* < 0.05, Mann–Whitney *U* test; Values shown are mean ± SEM

To validate the requirement of AMPAR and mTOR in antidepressant-like actions of echinacoside, we applied NBQX (an AMPAR inhibitor) and rapamycin (an mTOR inhibitor) before echinacoside administration, and then tested in the FST. Figure 3A presents the timeline of inhibitor pretreatment. As showed in Fig. 3B, the decreased immobility induced by echinacoside was completely reversed by NBQX and rapamycin. In addition, there is no significant difference between NBQX- or rapamycin-treated compared with saline-treated group. This confirms that echinacoside-induced antidepressant-like action required the activation of mTOR and AMPAR.

### Effects of AMPAR inhibitor NBQX and mTOR inhibitor rapamycin on the mTOR, Akt, and ERK activations caused by echinacoside

To elucidate the role of AMPAR and mTOR on the mTOR pathway-related activations caused by echinacoside, we analyzed the activations of Akt, and ERK, and mTOR signaling in echinacoside-treated mice with or without NBQX and rapamycin pretreatment. As showed in Fig. 3C, a single injection of echinacoside resulted in significant increases in the immunoreactions of pmTOR, pAkt, and pERK. The levels of total mTOR, Akt, and ERK remained unchanged. NBQX pretreatment blocked the echinacoside-induced increase in the levels of pmTOR (Fig. 3D), pAkt (Fig. 3E), and pERK (Fig. 3F). These data indicate that echinacoside-induced AMPAR activation is the upstream of Akt, ERK and mTOR signaling. In addition, the increased pmTOR levels induced by echinacoside was also completely abolished through pretreatment with rapamycin (Fig. 3D), but echinacoside-induced increases in pAkt and pERK were not attenuated by rapamycin pretreatment (Fig. 3E, F). These data indicate that echinacoside-induced mTOR activation is the downstream of Akt and ERK activations.

### Effects of ERK inhibitor SL327 and Akt inhibitor MK2206 on the decreased immobility caused by echinacoside in the mice FST

**Fig. 4:**
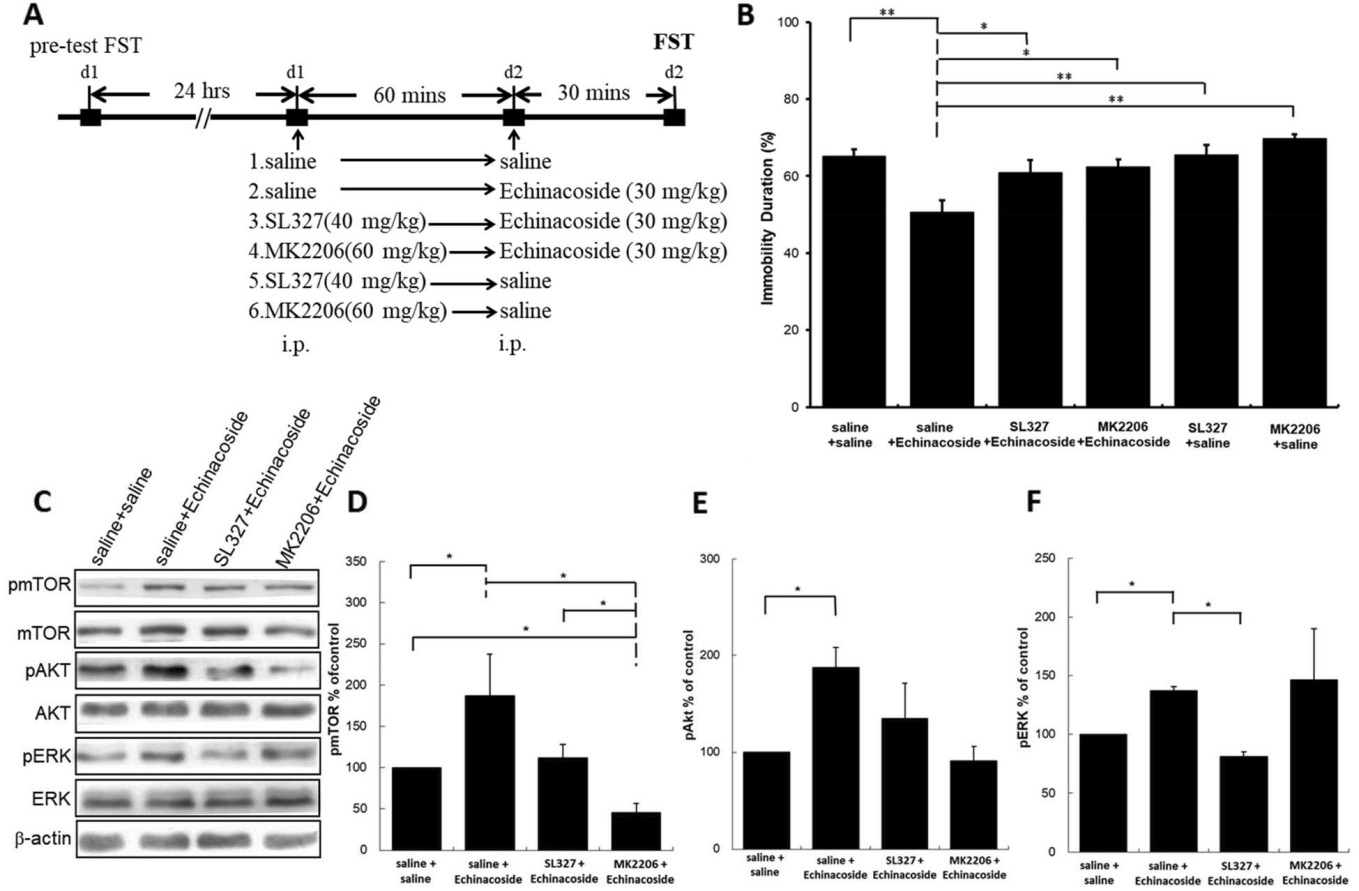
Effects of SL327 (40 mg/kg) or MK2206 (60 mg/kg) on the antidepressant-like effect and Western blotting of pmTOR, pAkt, and pERK of echinacoside (30 mg/kg)-treated mice. The timeline exhibits the experimental procedure under administration of drugs (**A**). Statistically analysis showed that the effect of reduction of immobility duration in FST resulted from echinacoside treatment is inhibited when the mice were pretreated with SL327 and MK2206 (**B**) (ANOVA, F(5.54)=7.195, p < 0.001; n=10 per group; *p < 0.05, **p < 0.01 compared with saline-treated group with Tukey *post hoc* analysis). Western blot analysis of phosphorylation of mTOR, Akt, and ERK was performed (**C**). The densitometry analyses of the blot (normalized to β-actin) confirmed the increased activity of pmTOR (**D**), pAkt (**E**), and pERK (**E**) in the echinacoside administrated group. Western blotting shows the increased expression of pmTOR caused by echinacoside treatment is blocked when the mice were pretreated with SL327 and MK2206 (**D**). The enhanced expression of pAkt (**E**) is blocked by pretreatment of MK2206, not SL327. The enhanced expression of pERK (**F**) is blocked by pretreatment of SLE 327, not MK2206. Total levels of Akt, ERK and mTOR were not different among the four groups. n = 4 each group; * p < 0.05, Mann–Whitney *U* test; Values shown are mean ± SEM

To validate the requirement of ERK and Akt in antidepressant-like actions of echinacoside, we applied ERK inhibitor SL327 and Akt inhibitor MK2206 before echinacoside treatment, and then tested in the FST. Figure 4A presents the timeline of inhibitor pretreatment. As showed in Fig. 4B, Echinacoside-induced antidepressant-like effects were blocked by SL327 and MK2206. And, no significant difference existed between SL327- or MK2206-treated compared with saline-treated group. This confirms that the antidepressant-like action of echinacoside depends on the activations of ERK and Akt signaling.

### Effects of ERK inhibitor SL327 and Akt inhibitor MK2206 on the mTOR, Akt, and ERK activations caused by echinacoside

To elucidate the role of Akt and ERK on the AMPAR–mTOR pathway activation caused by echinacoside, we measured pmTOR, pAkt, and pERK activations in the echinacoside-treated mice with or without SL327 and MK2206 pretreatment. As presented in Fig. 4C, SL327 (an ERK inhibitor) pretreatment blocked the echinacoside-induced increases of pmTOR (Fig. 4D) and pERK (Fig. 4F), but did not affect pAkt (Fig. 4E) immunoreactions; that confirmed the ERK is the upstream of mTOR activation. Moreover, Akt inhibitor MK2206 prevented the increase in pmTOR (Fig. 4D) and pAkt (Fig. 4E) engendered by echinacoside but did not modulate echinacoside-induced increase in pERK (Fig. 4F). It also confirmed the Akt is the upstream of mTOR activation. These results indicated that echinacoside could induce the activation of AMPAR–Akt/ERK–mTOR signal transduction, identical to that of ketamine [6].

### Modulations of AMPAR membrane insertion following echinacoside treatment

**Fig. 5:**
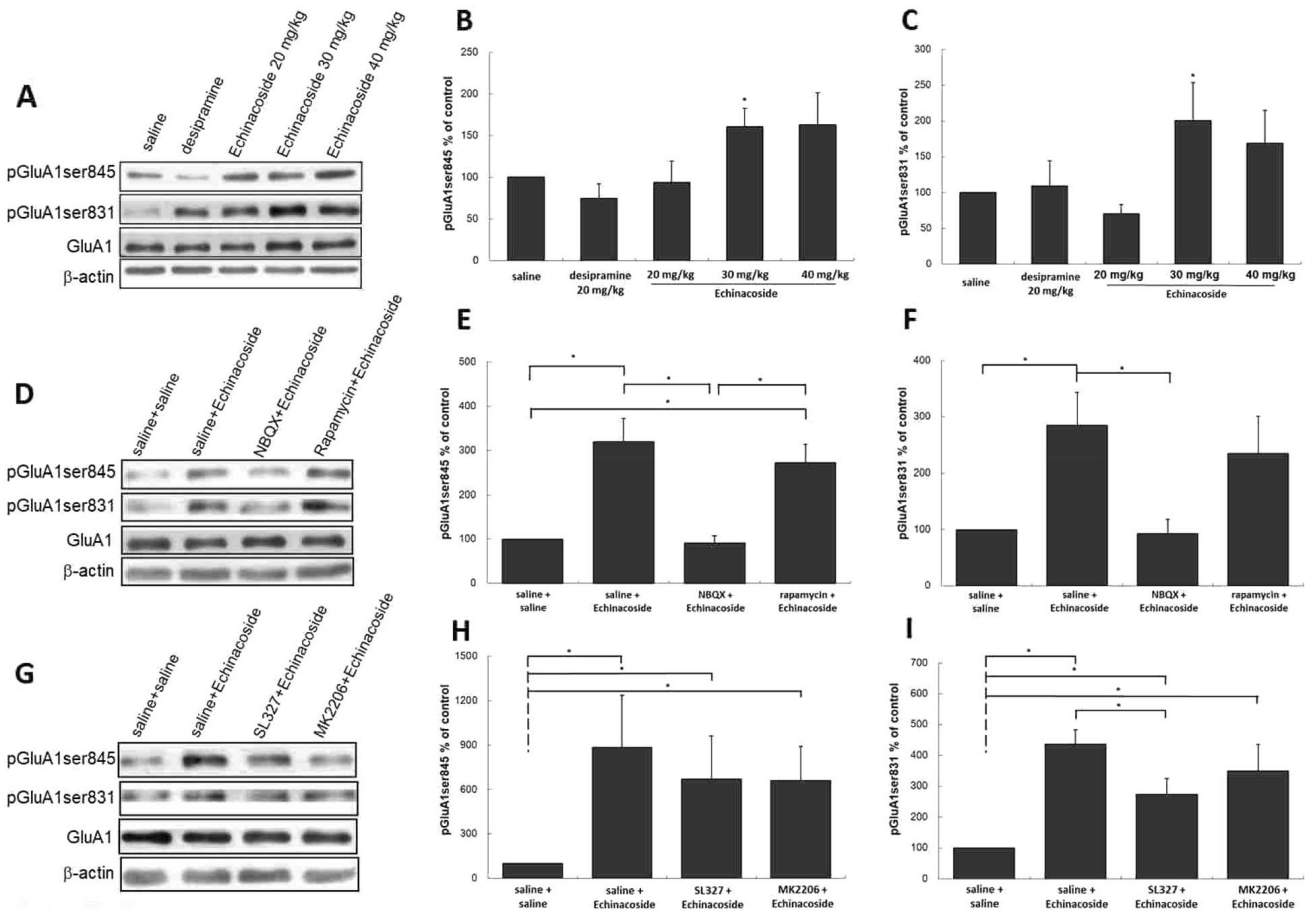
Representative Western blotting of pGluA1ser845 and pGluA1ser831 from hippocampus of mice treated with saline, desipramine (20 mg/kg) or echinacoside (20, 30 or 40 mg/kg) (**A**), after echinacoside (30 mg/kg) administration with pretreatment with NBQX (30 mg/kg) or rapamycin (20 mg/kg) (**D**) and after echinacoside (30 mg/kg) administration with pretreatment with SL327 (40 mg/kg) or MK2206 (60 mg/kg) (**G**). Echinacoside at 30 mg/kg treatment significantly increases the expression of pGluA1ser845 (**B**) and pGluA1ser831 (**C**), which were not observed in mice with other treatments. The densitometry analyses of the blot (normalized to β-actin) verify the enhanced activities of pGluA1ser845 (**E**), and pGluA1ser831 (**F**) in the echinacoside at 30 mg/kg administrated group and decreased activities in the NBQX, but, not in rapamycin pretreated groups (**D**–**F**). The up-regulations of pGluA1ser845 and pGluA1ser831 were not blocked by pretreatment of SL327 or MK2206 (**G**–**I**). n = 4 each group; *p < 0.05, Mann–Whitney *U* test; Values shown are mean ± SEM

AMPAR membrane insertion is also considered to be involved in the antidepressant effects of several new-generation glutamate-based antidepressants [15, 19–21]. Thus, we investigated whether echinacoside modulates the activated forms of the AMPAR subunit of GluA1 on its PKA (GluA1ser845) [22–24] and PKC (GluA1ser831) [25, 26] sites to assess the influence of echinacoside on the insertion of the AMPAR membrane. As shown in Fig. 5A, increases in the phosphorylation of GluA1ser845 and GluA1ser831 were observed in mice treated with 30 mg/kg echinacoside, which was not observed in mice treated with 20 mg/kg echinacoside and desipramine (Fig. 5B, C). Although 40 mg/kg echinacoside upregulated pGluA1ser845 and pGluA1ser831, the increase was not significant.

NBQX treatment before echinacoside reversed echinacoside-induced increases in the levels of pGluA1ser845 and pGluA1ser831 (Fig. 5E, F). However, treatment with rapamycin 30 min before echinacoside did not attenuate the echinacoside-induced increase. In addition, SL327 or MK2206 pretreatment before echinacoside did not significantly attenuate echinacoside-induced increases in both (Fig. 5H, I). The total levels of GluA1 remained unchanged following treatments (Fig. 5A, D, G). These data indicate that echinacoside could enhance AMPAR membrane insertion, which required AMPAR activation.

### Effect of echinacoside on BDNF expression

**Fig. 6:**
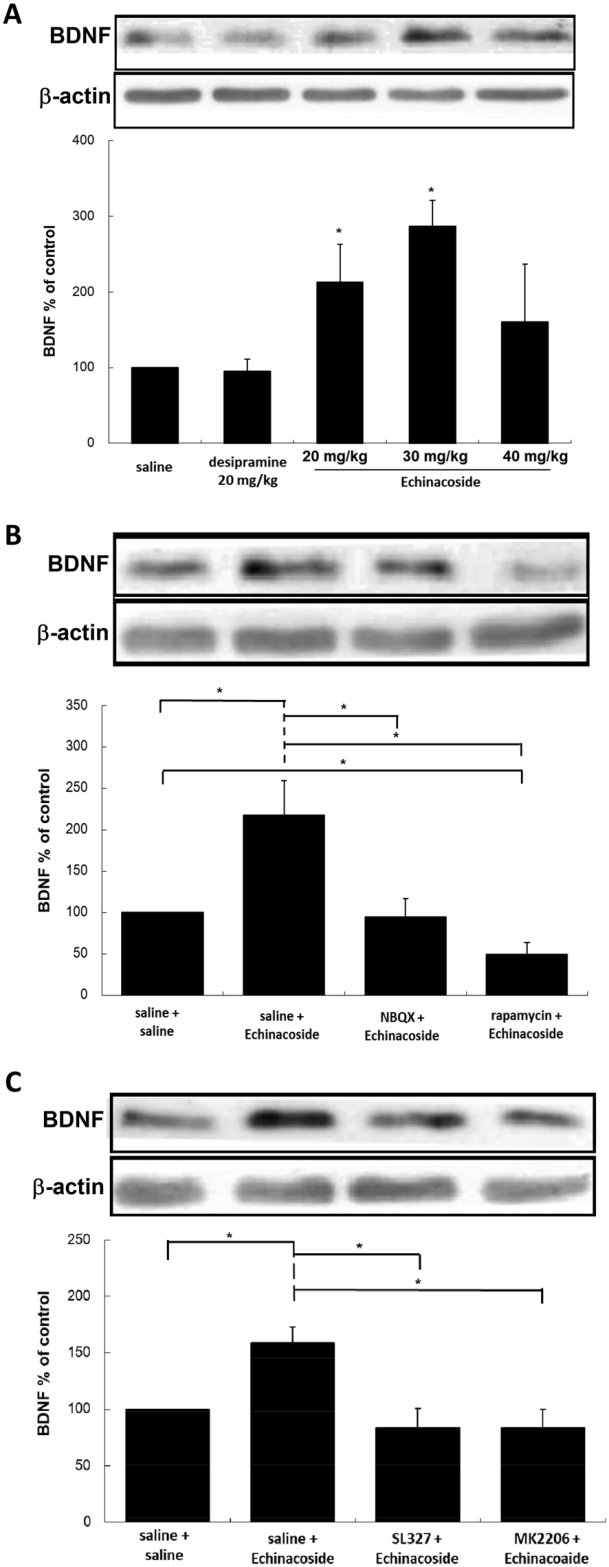
Representative Western blotting of the expression of BDNF from hippocampus of mice treated with saline, desipramine (20 mg/kg) or echinacoside (20, 30 or 40 mg/kg) (**A**), after echinacoside (30 mg/kg) administration with pretreatment with NBQX (30 mg/kg) or rapamycin (20 mg/kg) (**B**) and after echinacoside (30 mg/kg) administration with pretreatment with SL327 (40 mg/kg) or MK2206 (60 mg/kg) (**C**). Echinacoside at 20, 30 mg/kg treatment significantly increases the expression of BDNF (**B**), which was not observed in mice with other treatments. The densitometry analyses of the blot (normalized to β-actin) verify the enhanced expression of BDNF (**B**) in the echinacoside at 30 mg/kg administrated group and decreased activities in the NBQX and rapamycin pretreated groups (**B**). The up-regulation of BDNF was also blocked by pretreatment of SL327 or MK2206 (**C**). n = 4 each group; *p < 0.05, Mann–Whitney *U* test; Values shown are mean ± SEM

BDNF is a type of neurotrophin, which also plays a key role in neuronal development, differentiation, survival, and emotional regulation [27]. Moreover, BDNF released through ERK signaling results in a rapid antidepressant response [28]. We examined BDNF expression in mice treated with drugs. As presented in Fig. 6A, 20 and 30 mg/kg echinacoside treatment significantly upregulated BDNF expressions in the hippocampus; by contrast, desipramine and 40 mg/kg echinacoside did not affect the BDNF expressions. The trend was consistent with mTOR activation and antidepressant-like action in the FST. In addition, NBQX and rapamycin treatments before echinacoside blocked echinacoside-induced increase in BDNF (Fig. 6B). Finally, SL327 and MK2206 completely inhibited echinacoside-induced increases in BDNF (Fig. 6C).

## Discussion

Given the known effects that echinacoside increases the activation of Akt and ERK signaling in the brain [9, 10], we investigate whether echinacoside can provide antidepressant-like effects via increased Akt/ERK downstream mTOR signaling in the hippocampus. We confirmed that 30 mg/kg echinacoside could increase the activated Akt and ERK and found echinacoside provided antidepressant-like effects in the FST, accompanied by a significant increase of Akt/ERK downstream mTOR activation. Using pharmacological inhibitions, we subsequently observed that the antidepressant-like effects of echinacoside required the activation of the AMPAR–Akt/ERK–mTOR pathway, which is same as that of ketamine [6]. Moreover, echinacoside facilitated AMPAR membrane insertion and BDNF expression. These findings suggest that echinacoside is a potential therapeutic agent with rapid-onset antidepressant effects.

Echinacoside exhibits neuroprotective, antioxidant, anti-inflammation effects and therapeutic effects against Alzheimer and Parkinson diseases [8]. Because oxidative stress, inflammation, and these neurodegenerative diseases are highly correlated with depression, these reports imply the possibility of antidepressant-like actions of echinacoside [29, 30]. In addition, as evidence suggesting that echinacoside can upregulate brain Akt and ERK signaling [9, 10], their downstream mTOR signaling is suggested as a convergent mechanism of fast-acting antidepressant effects and closely related to depression pathophysiology [6, 31–33]. Accordingly, echinacoside was studied in this study. We used FST to examine antidepressant-like effects of echinacoside at different doses. We found that a single injection of 20, 30, or 40 mg/kg echinacoside could induce significantly reduced immobility in the FST without alteration of the locomotor activity in the OFT, verifying that echinacoside has antidepressant-like effects. We also found echinacoside exhibited an inverted U-shaped dose–response relationship in decreased immobility in the FST. Reduction in immobility time was more pronounced with 30 mg/kg echinacoside treatment than with 20 or 40 mg/kg echinacoside treatment; furthermore, 30 mg/kg echinacoside treatment significantly increased all immunoreactivities at the pAkt, pERK, pmTOR, and BDNF levels. The behavioral actions of echinacoside in the FST occurred in a nonlinear dose-response manner, parallel in the inducted intensities of pmTOR and BDNF expression. The reason for the inverted U-shaped dose–response relationship with echinacoside in behavioral actions and downstream pmTOR and BDNF remains unclear. But, this U-shaped dose–response phenomenon is not uncommon in pharmacological studies [34]. On the contrast, the effect of echinacoside on the upstream signaling, including pAkt and pERK, followed a linear or sigmoidal dose-response curve. These results indicate a floor/ceiling effect, even decreased efficacy, may occur on mTOR activation and BDNF when treated with echinacoside at high doses.

Subsequently, we elucidated the antidepressant-like mechanism of echinacoside. To examine whether the antidepressant-like response to echinacoside is mediated through mTOR activation directly, we administered the mTOR inhibitor rapamycin before echinacoside; then, we observed the behavior of the mice in the FST. We found that a pharmacological block of mTOR activation by rapamycin inhibited the antidepressant effects of echinacoside, verifying that echinacoside’s antidepressant-like effects depend on mTOR activation. To date, data published on the effect of echinacoside on mTOR signaling are limited to two in vitro studies for bone and intestine cells, and their results are contradictory [35, 36]. The first study found that echinacoside applied at doses of 10^−8^ to 10^−6^ M could promote mTOR activation in high-glucose-injured osteoblastic MC3T3-E1 cells [35]. By contrast, in the other in vitro study, echinacoside administered at 10 and 20 µg/mL inhibited mTOR activation caused by lipopolysaccharide in rat intestinal IEC-6 cells [36]. Although studies have revealed that echinacoside facilitates Akt and ERK production in the brain, neither an in vitro nor in vivo investigation has studied the effects of echinacoside on Akt/ERK downstream mTOR activation in the brain. Our study is the first in vivo study to demonstrate that echinacoside could significantly increase the activation of hippocampal mTOR signaling. In addition, the mTOR inhibitor rapamycin blocked the antidepressant responses and mTOR activation caused by echinacoside, confirming that the activation of hippocampal mTOR is required in the antidepressant responses of echinacoside. Our findings are consistent with those reported for the activated mTOR on antidepressant effects in previous studies [6, 31–33]. Duman’s group were the first to discover that mTOR activation in brain is required for the rapid antidepressant mechanism of ketamine [6]; soon after that, mTOR became a major target in the studies of depression and antidepressant action. Subsequently, various studies confirmed the crucial role of mTOR in the treatment and pathophysiology of depression. Furthermore, many traditionally used antidepressants (escitalopram, fluoxetine, and paroxetine) also have effects of increased mTOR activation [37]. Our results indicate the possible fast-acting benefit effect of echinacoside in depressed patients.

Meanwhile, the AMPAR inhibitor NBQX pretreatment reversed the antidepressant-like effects and the increases of activated mTOR and both mTOR upstream regulator kinases, Akt and ERK in echinacoside-treatment mice. These results confirmed that mTOR signaling activation through AMPAR participates in the antidepressant-like responses of echinacoside. Finally, regarding the Akt/ERK-mediated mechanisms of echinacoside, our study also revealed that hippocampal Akt and ERK expressions were upregulated after echinacoside treatment. The present results confirm those of previous reports [9, 10]. In addition, the blocking of Akt or ERK inhibited the antidepressant-like effect. The results also demonstrated that Akt and ERK are involved in the antidepressant-like actions of echinacoside. The antidepressant-like responses of echinacoside on Akt/ERK signaling occur during AMPAR and mTOR signaling downstream and upstream, respectively. Taken together, the results indicate that the antidepressant actions of echinacoside depend on the activations of AMPAR–Akt/ERK–mTOR signal transduction, which are identical to those of antidepressants with fast-acting property [6, 7, 33].

AMPAR, a subtype of ionotropic glutamate receptor, consists of GluA2 and GluA1, GluA3, or GluA4 subunits [38]. GluA1 is centrally involved in synaptic plasticity [39]. AMPAR trafficking is also crucial for antidepressant and neuroprotective effects [15, 19–21, 40]. To further investigate the effects of echinacoside on AMPAR trafficking, we analyzed the phosphorylation of AMPAR subunits GluA1 PKA (GluA1ser845) and GluA1 PKC (GluA1ser831), both of which are indicators of GluA1 membrane insertion [22–26]. Our immunohistochemical analyses demonstrated that the in vivo treatment with echinacoside increased AMPAR phosphorylation at GluA1ser845 and GluA1ser831. Our data verified that echinacoside can facilitate AMPAR insertion to the synapses, which can increase postsynaptic AMPAR levels, thus leading to the increase in the AMPAR:NMDAR ratio. Subsequently, increased AMPAR throughput activated mTOR, and finally, echinacoside provided antidepressant-like effects. These results indicate the antidepressant properties of echinacoside and are also correlated with those of ketamine, which also can increase GluA1 insertion into the postsynaptic membrane [41, 42]. The increased AMPAR throughput has been found in several antidepressants and proposed as a convergent response [43, 44].

Early studies have reported that echinacoside benefits human health, such as with neuroprotective effects in Parkinson and Alzheimer diseases [8]. Mitogen-activated protein kinase, NF-kappa B, caspase 3 and 8, and reactive oxygen species/activating transcription factor 3/C/EBP-homologous protein pathways have been proposed as the underlying neuroprotective mechanisms of echinacoside [8]. However, the actual mechanism remains unclear. Our in vivo study is the first to show that echinacoside induces AMPAR membrane insertion. Our findings suggest an alternative potential cellular mechanism to explain how echinacoside exerts its neuroprotective effect. Following AMPAR insertion facilitation, echinacoside regulates the expression of synapse-related proteins and potentially protects against maladaptive neurosynaptic deficit and cellular damage, resulting in neuroprotective effects in some diseases, particularly those of malalterations in synaptic plasticity.

A study reported that echinacoside exerts neuroprotective effects through the activation of the Trk/ERK signaling pathway, and BDNF was involved in this reaction [10]; furthermore, another study found that echinacoside treatment can reduce dopaminergic function and increase BDNF mRNA expression and its protein [45]. In addition to neuroprotection, BDNF plays a crucial role in depression and the therapeutic mechanisms of antidepressants [46]. To understand whether the antidepressant response of echinacoside is accompanied by BDNF variation, we examined the expression of BDNF through Western blotting. We found that echinacoside could upregulate BDNF expression. This finding is consistent with previous studies and is also relevant to the antidepressant effect of echinacoside. The same properties of echinacoside and ketamine affect the BDNF expression. However, the inhibitors of AMPAR, Akt, ERK, and mTOR could block BDNF expression induced by echinacoside, suggesting that BDNF expression is downstream of AMPAR–Akt/ERK–mTOR pathway signaling. The action is different from that of ketamine. Studies have indicated that ketamine can enhance glutamate release and block NMDAR, which activate another glutamate receptor, AMPAR; this in turn triggers BNDF release, which causes downstream mTOR activation [46]. Moreover, we suggest that BDNF plays a role in the antidepressant-like properties and AMPAR–Akt/ERK–mTOR signaling pathway activation caused by echinacoside, although future mechanistic studies of this interaction are required.

## Conclusions

Our results verified that the natural phenol echinacoside exhibits antidepressant-like effects through the activation of the AMPAR–Akt/ERK–mTOR signaling pathway, which is similar to that of ketamine. Moreover, echinacoside enhances AMPAR membrane insertion and BDNF expression, indicating that echinacoside potentially provides neuroprotection by triggering AMPAR membrane insertion and BDNF upregulation. Our findings serve as preclinical evidence that echinacoside can target the hippocampal glutamatergic pathway and act as a rapid-acting antidepressant.

## Supplementary Information

**Additional file 1.** The information of Western blot analysis used in this study.

## Data Availability

The data can be requested from the author upon reasonable request.

## Abbreviations

NMDAR: *N*-methyl-d-aspartate
AMPAR: α-Amino3-hydroxy-5-methyl-4-isoxazolepropionic acid receptor
BDNF: Brain-derived neurotrophic factor
Akt: Protein kinase B
ERK: Extracellular signal–regulated kinase
mTOR: Mechanistic target of rapamycin
FST: Forced swimming test
OFT: Open field test
GluA1ser845: AMPAR subunit of GluA1 on PKA site
GluA1ser831: AMPAR subunit of GluA1 on PKC site
