# Supplementary material for: Echinacoside exhibits antidepressant-like effects through AMPAR–Akt/ERK–mTOR pathway stimulation and BDNF expression in mice
Source: Chin Med. 2022 Jan 5;17:9. doi: 10.1186/s13020-021-00549-5 (PMC8728918; doi:10.1186/s13020-021-00549-5)
Supplement: Supplementary file 1 — Additional file 1. The information of Western blot analysis used in this study. [file 13020_2021_549_MOESM1_ESM.doc]

**Echinacoside exhibits antidepressant-like effects through AMPAR–Akt/ERK–mTOR pathway stimulation and BDNF expression in mice**

***Supplemental information***

**Western Blot analysis**

Previously described procedures for Western blot analysis followed . Solubilized proteins (100μg) were electrophoretically separated in the 10% SDS polyacrylamide gel, transferred to nitrocellulose membranes, and then they were stained with Ponceau Red to confirm equal protein loading. The nitro-cellulose membranes were blocked by 5% skimmed milk in Tris-buffered saline solution containing 0.1% Tween 20 at room temperature for 1 hour. To detect expression level of specific protein, the membrane were probed with anti-phospho-mTOR (1:1000, Cell Signaling), anti-phospho-AMPAR GluA1 (1:1000, at ser845 and ser831, Sigma), anti-phosphor-Akt (1:1000, at ser-473, Millipore), anti-phospho-ERK (1:1000, at Thr185/Tyr187 and Thr202/ Tyr204, Millipore), BDNF (1:500, Santa Cruz Biotechnology) at 4°C overnight and relatively probed with anti-β-actin (1:2000, Millipore), anti-total mTOR (1:1000, Millipore), anti-total Akt (1:1000, Cell Signaling), anti-total ERK (1:1000, Millipore) or anti-AMPAR GluA1 (1:1000, Millipore) primary antibodies as controls. The membrane were further incubated with horseradish peroxidase(HRP)-conjugated secondary antibody (1:1000; Santa Cruz Biotechnology) for 1.5 hours at room temperature after washing unbound antibodies with TBST buffer. Then we developed the membranes with enhanced chemilluminescence kit (Millipore) and exposed onto Kodak MR Film. The optic densities were measured and quantified by Gel-Pro analyzer 3.0 computer-assisted program.

1. Chen KT, Tsai MH, Wu CH, Jou MJ, Wei IH, Huang CC**: AMPA Receptor-mTOR Activation is Required for the Antidepressant-Like Effects of Sarcosine during the Forced Swim Test in Rats: Insertion of AMPA Receptor may Play a Rol**e*. Front Behav Neurosc*i 2015**,** 9:162.
